# Psychosocial Risks, Work Engagement, and Job Satisfaction of Nurses During COVID-19 Pandemic

**DOI:** 10.3389/fpubh.2020.566896

**Published:** 2020-11-20

**Authors:** María del Carmen Giménez-Espert, Vicente Prado-Gascó, Ana Soto-Rubio

**Affiliations:** ^1^Department of Nursing, Faculty of Nursing and Chiropody, University of Valencia, Valencia, Spain; ^2^Social Psychology Department, Faculty of Psychology, University of Valencia, Valencia, Spain; ^3^Personality, Assessment and Psychological Treatments Department, Faculty of Psychology, University of Valencia, Valencia, Spain

**Keywords:** COVID-19, psychosocial risks, work engagement, job insecurity, nurse, peak pandemic, job satisfaction

## Abstract

**Context:** COVID-19 pandemic is a serious health emergency that has affected countries all over the world. Health emergencies are a critical psychosocial risk factor for nurses. In general, psychosocial risks constitute serious problems as they impact workers' health, productivity, and efficiency. Despite their importance, few studies analyze nurses' psychosocial risks during a health emergency caused by a pandemic or analyze their perception of the emergency and its relation to such risks.

**Objectives:** To analyze the perception of COVID-19 by nurses, especially about measures, resources, and impact on their daily work. Also, to analyze these professionals' psychosocial risks and the relationship between perception of COVID-19 and these risks.

**Methods:** A descriptive correlational study was performed in a convenience sample of 92 nurses from two public hospitals in the Valencian Community (Spain), (74 women, 79.1%), aged 24–63 (M = 43.37, SD = 11.58). Data were collected via an online self-completed questionnaire during the rise of the pandemic from March 29 to April 8, when the number of infections went from 78,797 to 146,690.

**Results:** The measures and resources available about COVID-19 are relatively low, and the impact on their work is high. Similarly, the most prominent psychosocial risks appear to be emotional work and workload. In contrast, nurses' work engagement is medium, and their satisfaction is high. Finally, there seems to be a negative and significant relationship between the information available to nurses, the measures implemented, and resources with some of their psychosocial risks, and a positive one with job satisfaction and work engagement. There is also a positive and significant relationship only between the impact of COVID-19 and their work inequality, but not for other risks.

**Conclusions:** The resources, measures, and information can be a protective factor facing nurses' psychosocial risks, especially during a pandemic. Studying the relationships between psychosocial risk and perception of a health emergency would be relevant and fundamental to protecting and caring for nurses, health professionals, and society.

## Introduction

Psychosocial risks at work are aspects of work design and the social, organizational, and management contexts of work that could cause psychological or physical harm (1). Psychosocial risks and work-related stress are among the most challenging issues in occupational safety and health, impacting significantly on the health of individuals, organizations, and national economies (2, 3). They arise from inadequate work design, management, organization, and poor social context of work, resulting in adverse physical, psychological, and social outcomes such as work-related stress, depression, or burnout (4). More specifically, psychosocial risks are related to low job satisfaction (5), health problems (3), work accidents (6), work-related stress (7), burnout (8). Psychosocial risks are closely related to work-related stress, which has been associated with a reduction in social interaction and the ability to concentrate at work, increased physiological pain and cardiovascular problems, and a higher incidence of mental illness such as depression and anxiety (9, 10). Stress, and the psychosocial risks that can exacerbate it, could also affect other aspects of work such as job satisfaction and motivation (11) or work engagement (12). In this same vein, the proper management of psychosocial risk helps to prevent accidents and absenteeism (5, 13), increase productivity (5, 14, 15), and promote well-being at the workplace (16).

Among the different sectors, the health sector is the one that traditionally seems to be most affected by these types of conditions, in particular concerning physicians and nurses, who constitute a professional group that meets high responsibility, work demands, and job insecurity; and, at the same time, a great commitment to their work (17).

This data is of paramount importance, since nurses play a vital role in the health systems, constituting the largest group of health professionals (18). Nurses' contribution to global health is undisputed, and investing in improving their quality of life benefits society (19, 20). Improved working conditions and professional development affect not only the well-being or quality of life of nurses but also their performance and the functioning of the entire health care system (21). In line with this, as the WHO suggests, adequate staffing and prioritization of occupational health and safety are essential (18).

Among the different theoretical models that exist to explain the appearance of occupational stress, Karasek's model (22) is the one with the most theoretical and empirical support and the one that currently has the most influence and attention. It explains work-related stress according to the imbalance between psychology demands at work (e.g., workload, role conflicts, interpersonal conflicts, job insecurity) and the control level or resources that the employee has. According to this model, the employees' health or well-being depends on balancing their work demands and their own resources. When the demands are higher than the resources, it can feel like work-related stress by the employee. In addition, chronic work-related stress can cause burnout syndrome and several physical or psychosomatic symptomatologies. Thus, an excess of demands will produce a negative consequence in the employee, as higher burnout, however having enough resources benefit the employee decreasing the probability of having higher burnout (23).

Among the different psychosocial risks, the following stand out because of their importance:

Role conflict: This is the situation in which a worker cannot simultaneously satisfy the contradictory role expectations in which he or she is involved. There is role conflict when a worker receives contradictory demands from two or more people, or tasks without having the necessary resources to complete them. Previous research has shown that problematic distress levels were 53 percent more likely for workers reporting role conflict (24).

Lack of organizational justice: Lack of organizational justice refers to the extent to which employees perceive they are treated unfairly in their workplace and the perception of the absence of reciprocity in social exchanges (25). Low organizational justice is known to be a potential risk factor for poor physical and psychological health among employees (25).

Workload: It applies to quantitative and qualitative workload. Quantitative workload refers to the number of activities to be performed in a given time. In contrast, qualitative workload refers to the difficulty of the task and the volume of information to be processed in relation to the time available (26). A high workload has been associated with low well-being and high risks of health problems (27).

Interpersonal conflicts: It refers to the frequency with which workers perceive that conflicts are coming from the hospital management, colleagues, patients, or relatives of the patient. Interpersonal conflicts have been associated with health problems, particularly depression (28).

Emotional work: It refers to the effort, planning, and control necessary to express the organizationally desirable emotions during interpersonal transactions (29). It includes emotional demands, such as “dealing with strong feelings such as sorrow, anger, desperation, and frustration” at work (24). Previous research has shown that problematic distress levels were 38 percent more likely for workers reporting high emotional work (24).

Job insecurity: is the perceived threat of losing one's current job in the near future (30), or also that the employer did not comply with his obligations or promises (breach of psychological contract) (31), which can have equally severe consequences as actual job loss (32). Particularly, job insecurity is considered a stressor that negatively affects the employee's physical, psychological, and social health (33–35).

Among the most critical consequences of psychosocial risk factors are psychosomatic health problems and burnout syndrome.

Psychosomatic health problems: The term psychosomatic refers to alterations in which mental processes influence the organism (36). Among the most common are various types of symptoms affecting multiple organs and systems. Examples of these are back pain, tension headaches, sleep problems, chronic fatigue, heartburn, tension diarrhea, or heart palpitations (37).

Burnout syndrome: is defined as a prolonged response to chronic emotional and interpersonal stressors at work and is defined by the three dimensions of burnout, cynicism, and inefficiency (9).

Although most of the available studies on psychosocial risks tend to focus on their negative consequences or outcomes such as stress, psychosomatic problems, or burnout, psychosocial risk management also has positive outcomes. Job satisfaction and work engagement are among these positive outcomes.

Job satisfaction: It can be described as how much people like or dislike their jobs (38) or how much they perceive their needs met by work (39). There is a consensus among the several models that explain job satisfaction: it is influenced by external factors such as working conditions and internal factors such as self-efficacy beliefs (40).

Work engagement: it presents three dimensions (1) Dedication, defined by feelings of importance, inspiration, challenge, enthusiasm and pride; (2) Vigor, defined by a high level of energy and mental stamina at work, eagerness to put effort into one's work, and determination to overcome challenges; and (3) Absorption, defined by being completely focused and deeply immersed in one's work, so that time passes fast and one has difficulty letting go (41).

Work engagement can be differentiated from other types of worker well-being, such as burnout, boredom, work addiction, and job satisfaction. Work engagement has been conceived as the opposite and positive pole of burnout, characterized by mental fatigue related to work (9). As a result, burnout and work engagement relate negatively. Boredom at work, like burnout, is defined by little excitement and displeasure (42), while work engagement is defined by great excitement and pleasure. Work engagement can also be differentiated from work addiction, which applies to a strong inner compulsion to excessive work (43), defined by high excitement and displeasure. Work engagement can also be distinguished from job satisfaction (44). Although both are defined by pleasure, the degree of enthusiasm for engagement is higher than for job satisfaction (45).

Working conditions, and the consequences that arise from them, can be significantly affected by the economic and social context (46), especially when events that affect the entire population arise, such as economic crises or, in this case, health emergencies or pandemics, such as that caused by COVID-19.

The World Health Organization (WHO) recognized it as a global pandemic on March 11, 2020 (47). As of May 17, 2020, more than 4.8 million cases of the disease have been reported in more than 213 countries and territories worldwide, with nearly 316,000 deaths and more than 1.8 million recoveries (48, 49). The five countries with the highest number of infections are the United States, Russia, Brazil, the United Kingdom, and Spain (48, 49). The five countries with the highest number of deaths are the United States, the United Kingdom, Italy, France, and Spain (48, 49).

Public health emergencies affect the health, safety, and well-being of individuals. They usually generate confusion, insecurity, emotional isolation, and stigma. Public health emergencies also affect communities, leading to work and school closures, economic loss, and medical response resources scarcity. These effects may translate into a range of emotional reactions like distress or psychiatric conditions, unhealthy behaviors like substance abuse, and non-compliance with public health directives such as home confinement and vaccination (50).

The work of nurses involves several specific demands that make this group particularly vulnerable to psychosocial risks. This situation is even more dangerous in a pandemic situation such as that triggered by COVID-19, in which there is a massive increase in work demands.

Health care providers are particularly vulnerable to emotional distress in the current pandemic, given the novel nature of SARS-CoV-2 and their risk of exposure to the virus, increased workload, scarcity of personal protective equipment and other medical supplies, inadequate testing, limited treatment options, concern about infecting and caring for their loved ones, and involvement in emotionally and ethically fraught resource-allocation decisions (50).

In Spain, the alarming health situation generated by the COVID-19 pandemic has meant enormous overexertion of all health personnel at the national level, including nurses, who have had to face physical, psychological, emotional, and social demands in a situation where resources are not always available, and the uncertainty of the evolution of the pandemic has been present. Supplies of personal protective equipment in health centers have been a concern in all regions leading to re-use, despite the known risks (51). Many reports suggest that health care staff are stretched to the point of exhaustion, and the problems are being intensified by the quarantining of an increasing number of health workers (51). Insufficient measures have been taken, such as canceling holidays, bringing retired nurses, and doctors back into the health service, hiring graduates without specialization hiring final year medical and nursing students, and extending contracts of medical residents (51).

In Spain, as of May 17, 2020, there have been 231,606 confirmed cases, of which 150,376 have been discharged, 125,233 have been hospitalized, 11,437 have been admitted to Intensive Care Units (ICUs), and 27,709 have died, according to official data from the Ministry of Health (52).

In Spain, the first positive diagnosis was confirmed on January 31, 2020, on the island of La Gomera (53), while the first death occurred on February 13 in Valencia, a fact known 20 days later (54).

Given the rapid spread of the virus, on March 14, the Spanish government decreed an emergency state throughout the country for fifth teen days (55). This measure restricts citizens' free movement to some instances, such as purchasing food and medicines or visiting medical centers or the workplace. In practice, it confines the population to their place of residence. Since then, the Deputies Congress has authorized the government to extend the state of emergency on five occasions, extending this measure until June 7 (56). The Spanish government approved on April 28 (57) a plan for asymmetric de-escalation by territorial units. During this time, one of the main peaks of the pandemic in Spain occurred between late March and early April. Data on the daily evolution of the pandemic in Spain according to the level of severity of those infected during late March and early April (52) are presented in Table 1.

**Table 1:** Daily evolution of the pandemic in Spain according to the level of severity of those infected.

	**New cases**	**Hospital admissions**	**ICUs**	**Deceased**
24-Mar	8,563	3,702	541	596
25-Mar	8,959	4,112	303	615
26-Mar	9,189	4,849	536	745
27-Mar	8,253	4,361	338	839
28-Mar	6,428	2,626	310	715
29-Mar	5,813	2,910	252	696
30-Mar	8,148	2,427	309	820
31-Mar	7,413	3,073	292	929
01-Apr	7,591	2,502	213	877
02-Apr	7,280	2,189	221	845
03-Apr	6,678	2,463	192	780
04-Apr	5,539	1,556	311	670
05-Apr	3,672	1,232	102	628
06-Apr	5,213	1,284	124	757
07-Apr	5,586	1,536	190	757
08-Apr	5,749	1,698	219	781
09-Apr	4,540	1,853	124	642

Along with the impact that a pandemic can have on its own, a key element is the pandemic's perception by those who live with it, especially frontline workers, the nurses. Their perception of the measures taken, the resources available, and the pandemic's impact on their work and lives can affect and be affected by psychosocial risks and their consequences.

Despite the impact of pandemics on citizens' health and well-being, and more specifically of their workers, and its clear influence on working conditions, or more specifically on their psychosocial risks, there are hardly any studies that have addressed the effect of a pandemic on psychosocial risks. This situation is even more limited if we consider the impact on nursing professionals.

Likewise, the few studies traditionally available have been carried out retrospectively, ignoring their perception of the pandemic and the associated psychosocial risks during the times of greatest severity, or peak. Similarly, as mentioned above, studies on psychosocial risks have focused more on negative consequences such as stress or burnout while ignoring others in a positive sense, such as work engagement.

After conducting a review of the literature, we were unable to observe any studies focused on nurses that analyzed the psychosocial risks and their perception of the pandemic during its peak, considering not only negative consequences such as psychosomatic problems but also positive aspects such as job satisfaction and work engagement.

Therefore, the study presented here aims to fill this gap in the literature by offering a first approach to the perception of COVID-19 by nursing professionals and its relationship with psychosocial risks and some of its main consequences, such as psychosomatic problems, job satisfaction, and work engagement during the peak of the pandemic in Spain from March 29 to April 8, 2020.

### Aims

To analyze the perception of COVID-19 by nurses, especially about measures, resources, and impact on their daily work. Also, to analyze these professionals' psychosocial risks and the relationship between perception of COVID-19 and these risks.

## Methods

### Design, Procedure, and Participants

Ninety-two nurses from two public hospitals in the Valencian Community (Spain). The participants' age range was 24–63 (M = 43.37, SD = 11.58), and 79.1% of them were women.

The eligibility criteria for participants were as follows.

Inclusion criteria:

(a) To be a nurse.
(b) To be actively working during the moment of assessment.
(c) To have signed the informed consent document and confidentiality agreement within the framework of the Declaration of Helsinki principles.

Data were collected online with a self-completed questionnaire during the rise of the pandemic from March 29 to April 8, 2020, when the number of infections went from 78,797 to 146,690. This study was authorized by the Ethical Committee of Research with Medicines CEIM Code 128/19. The hospitals nursing units contacted the possible participants via email, and invited them to participate in the study. The time of completion of the entire assessment protocol was 45 min.

### Outcome Measures

The study involved the following variables and measurement tools:

*(a) Psychosocial risks*. Different scales have been used to measure demand and consequence factors.

Within the demand factors, we find:

Role conflict: Included in the UNIPSICO battery (26). Role conflict is the situation in which a worker cannot simultaneously satisfy the contradictory role expectations in which he or she is involved. The scale comprises 5 items (“I receive incompatible demands from two or more people”). It is answered on a 4-point Likert scale (0 = Never; 4 = Very frequently: every day), with higher scores indicating higher role conflict (scores above 1.6 are considered high, whereas scores equal or below 0.81 are considered as low). The alpha de Cronbach for the sample of study is α = 0.78.

Lack of organizational justice: Included in the UNIPSICO battery (26). Lack of organizational justice is defined as the perception of the absence of reciprocity in social exchanges. The scale is made up of 5 items (“I give up my skin at work compared to what I receive in return”). It is answered on a 4-point Likert scale (0 = Never; 4 = Very frequently: every day), with higher scores indicating a higher lack of organizational justice (scores above 2.4 are considered as high, whereas scores equal or below 1.6 are considered as low). The alpha de Cronbach for the sample of study is α = 0.88.

Workload: Included in the UNIPSICO battery (26), it assesses quantitative and qualitative workload. Quantitative workload refers to the number of activities to be performed in a given time. In contrast, qualitative workload refers to the difficulty of the task and the volume of information to be processed in relation to the time available. It consists of 6 items, 3 of quantitative (Is it possible for you to work at a relaxed pace?) and 3 of qualitative (When you are working, do you encounter particularly hard situations?). It is answered on a 4-point Likert scale (0 = Never; 4 = Very frequently: every day), where higher scores indicate a higher workload (scores above 2.17 are considered high, whereas scores equal or below 1.51 are considered low). The alpha de Cronbach for the sample of study is α = 0.77.

Interpersonal conflicts: Included in the UNIPSICO battery (26), it assesses the frequency with which workers perceive conflicts coming from the hospital management, colleagues, patients, relatives of the patient. The scale consists of 6 items (how often do you have conflicts with your colleagues?). It is answered on a 4-point Likert scale (0 = Never; 4 = Very frequently: every day), with higher scores indicating higher Interpersonal conflicts (scores above 1 are considered high, whereas scores equal or below 0.6 are considered low). The alpha de Cronbach for the sample of study is α = 0.43.

Emotional work: An adaptation of the Frankfurt Emotion Work Scales (FEWS) questionnaire (58) included in the UNIPSICO battery (26) has been used. This questionnaire defines emotional work as the effort, planning, and control necessary to express the organizationally desirable emotions during interpersonal transactions (29, 59). For the present study, 12 items were selected (Do you have to express pleasant emotions toward patients and their families? (e.g., kindness). It is answered on a 4-point Likert scale (0 = Never; 4 = Very frequently: every day). Higher scores indicate higher emotional work. The alpha de Cronbach for the sample of study is α = 0.56.

Job insecurity: It was measured using the Job Insecurity Scale (60). It consists of five items (“I feel insecure about the future of my job”) designed to measure quantitative job insecurity (i.e., insecurity to lose the job as such). Respondents were asked to rate these items on a 5-point Likert type scale, ranging from 1 (“strongly disagree”) to 5 (“strongly agree”), with higher scores indicating higher job insecurity levels. The alpha de Cronbach for the sample of study is α = 0.89.

Within the consequence factors, we find:

Psychosomatic problems. Included in the UNIPSICO battery (26), it assesses the frequency of occurrence of psychosomatic problems related to the perception of stress sources at work. It consists of 9 items related to the organism (e.g., “Have you been worried that, without making any effort, your breathing would be cut off?”). It is answered on a 4-point Likert scale (0 = Never; 4 = Very frequently: every day), with higher scores indicating higher Psychosomatic problems (scores above 1.67 are considered as high, whereas scores equal or below 0.89 are considered as low). The alpha de Cronbach for the sample of study is α = 0.88.

Job satisfaction. It is defined as a positive emotional state resulting from the person's work perception. This variable was measured using the job satisfaction scale of the UNIPSICO Battery (26), which contains a set of attitudes developed by the person toward specific facets of the job. It consists of 6 items (The opportunities offered by your job to do the things you like). Participants were asked to score the frequency with which they have experienced the situation described in each statement on a Likert type scale from 0 to 4 (0 = strongly unsatisfied; 4 = strongly satisfied) with higher scores indicating higher Job satisfaction. The alpha de Cronbach for the sample of study is α = 0.78.

Work engagement. To assess this variable the Ultra-Short Measure for work Engagement UWES-3 (45) was used, a shortened version of the Utrecht Work Engagement Scale or UWES (61). This scale includes three dimensions (41): (1) vigor, characterized by “high levels of energy and mental resilience while working, the willingness to invest effort in one's work, and persistence even in the face of difficulties”; (2) dedication, characterized by “feelings of a sense of significance, enthusiasm, inspiration, pride, and challenge”; and (3) absorption, characterized by “being fully concentrated and deeply engrossed in one's work, whereby time passes quickly, and one has difficulties with detaching oneself” (41). Respondents were asked to rate these items on a 5-point Likert type scale, ranging from 1 (“strongly agree”) to 5 (“strongly disagree”), with higher scores indicating higher work engagement. The alpha de Cronbach for the sample of study is α = 0.81.

*(b) COVID-19 related measures*. An *ad-hoc* questionnaire was constructed to measure different aspects related to the health emergency caused by the COVID-19. The aspects considered are Available resources (provided by the health center, regional government, and national government), information (provided by the health center, regional government, and national government), measures (taken by the health center, regional government, and national government) and impact on work (workload, labor conflicts, work-related stress, and work-related concerns and fears). The *ad-hoc* questionnaire includes 13 items, where the subject scores on a Likert scale his/her level of agreement or disagreement with the statements (1 = totally disagree, 5 = totally agree). Scores range from 1 to 5, with higher levels indicating greater satisfaction with the resources available, information, and measures taken, as well as higher levels of impact on work. The alpha de Cronbach for the sample of study is as follows: available resources α = 0.92; information α = 0.95; measures α = 0.92; impact on work α = 0.73.

### Data Analyses

A descriptive statistical analysis was performed for all study variables, as well as a study of correlations between them. All analyses were carried out using the IBM® SPSS® Statistics software (version 24).

## Results

### Descriptive Analysis

#### Psychosocial Risks and Their Consequences

As shown in Table 2, during the pandemic's peak, the perception of psychosocial risks was higher for Emotional Work and Workload than for the rest of the psychosocial risks, presenting Interpersonal conflicts problems the lowest scores. Regarding the consequences of psychosocial risks, scores on psychosomatic problems are low, and Job satisfaction, as well as work engagement, obtained scores slightly above the middle of the score range.

**Table 2:** Descriptive data of psychosocial risks and their consequences.

	**Mean**	**SD**	**Range**	**Risk level**
Role conflict	1.143	0.569	0–4	Medium
Lack of organizational justice	1.862	0.654	0–4	Medium
Workload	2.035	0.607	0–4	Medium
Interpersonal conflicts	0.692	0.462	0–4	Medium
Emotional work	3.437	0.398	0–4	–
Job insecurity	1.750	0.959	1–5	–
Psychosomatic problems	1.098	0.462	0–4	Medium
Job satisfaction	2.405	0.803	0–4	Medium
Work engagement	2.435	0.936	1–5	–

*M = Mean; SD = Standard deviation; Range 0–4 (0 = Never; 4 = Very frequently: every day); 1–5 (1 = “strongly disagree”; 5 = “strongly agree”); – not applicable*.

#### COVID-19 Related Measures

As shown in Table 3, during the pandemic's peak od, participants rated the resources available and measures taken by the government and the hospital slightly below the mean value of the answer scale, which points to a tendency to consider resources and measures an insufficient. Similarly, participants rated the information available regarding the pandemic slightly above the mean value of the answer scale, which points to a tendency to consider the information available as barely enough, but not satisfactory, which would have been closer to the top score of the answer scale. Finally, the mean of the scores on the workplace's impact is the highest among the COVID-19 related measures, being close to the top of the range of scores for this measure, which points to a high impact in general of the pandemic on the workplace.

**Table 3:** Descriptive data of COVID-19 related measures.

	**Mean**	**SD**	**Range**
Resources	2.256	1.045	1–5
Measures	2.444	1.073	1–5
Information	2.759	1.117	1–5
Impact on the workplace	3,873	0.862	1–5

### Analysis of Relations

The results of the correlation analysis among the variables are shown in Table 4. In regard to the psychosocial risks variables, note that job satisfaction correlates negatively with role conflict (*r* = −0.547; *p* < 0.01) and psychosomatic problems (*r* = −0.380; *p* < 0.01). Also role conflict correlates positively with interpersonal conflicts (*r* = 0.271; *p* < 0.05). Regarding the COVID-19 related variables, highlight that resources, measures and information correlate between them, in a very strong and positive way; particularly resources with measures (*r* = 0.839; *p* < 0.01) and measures with information (*r* = 0.776; *p* < 0.01). Nevertheless, none of this three variables correlated significantly with Impact of COVID-19. Finally, among the stronger correlations between variables of psychosocial risks and variables related to COVID-19, remark that resources (*r* = 0.474; *p* < 0.01), measures (*r* = 0.483; *p* < 0.01) and information (*r* = 0.558; *p* < 0.01) correlated positively with Job satisfaction. Also, resources (*r* = −0.312; *p* < 0.01), measures (*r* = −0.462; *p* < 0.01), and information (*r* = −0.529; *p* < 0.01) correlated negatively to Role conflict. In addition Workload correlates positive with Job insecurity (*r* = 0.292; *p* < 0.01), and psychosomatic problems (*r* = 0.369; *p* < 0.01), and negatively with job satisfaction (*r* = −0.364; *p* < 0.01) and COVID related resources (*r* = −0.271; *p* < 0.05), measures (*r* = −0.232; *p* < 0.05) and information (*r* = −0.408; *p* < 0.01).

**Table 4:** Correlations among the variables of study.

	**RC**	**LOJ**	**WL**	**IC**	**EW**	**JI**	**PP**	**JS**	**WE**	**R**	**M**	**INF**	**IMP**
Role conflict	1												
Lack of organizational justice	0.338^**^	1											
Workload	0.385^**^	0.476^**^	1										
Interpersonal conflicts	0.271^*^	−0.070	0.167	1									
Emotional work	0.101	0.229	0.115	−0.113	1								
Job insecurity	0.372^**^	0.184	0.292^*^	−0.066	−0.027	1							
Psychosomatic problems	0.591^**^	0.371^**^	0.369^**^	0.027	0.162	0.371^**^	1						
Job satisfaction	−0.547^**^	−0.226	−0.364^**^	−0.092	0.071	−0.246^*^	−0.380^**^	1					
Work engagement	0.280^*^	0.069	0.059	0.006	−0.029	0.087	0.031	−0.183	1				
COVID-19 resources	−0.312^**^	−0.191	−0.273^*^	0.007	−0.071	−0.030	−0.218	0.474^**^	0.125	1			
COVID-19 measures	−0.462^**^	−0.056	−0.232	−0.124	−0.006	−0.062	−0.255^*^	0.483^**^	0.047	0.839^**^	1		
COVID-19 information	−0.529^**^	−0.163	−0.408^**^	−0.129	−0.006	−0.265^*^	−0.250^*^	0.558^**^	−0.011	0.639^**^	0.776^**^	1	
COVID-19 impact	0.206	0.323^**^	0.282^*^	−0.043	0.186	0.177	0.234	−0.165	0.187	−0.176	−0.192	−0.176	1

RC, Role conflict; LOJ, Lack of organizational justice; WL, Workload; IC, Interpersonal conflicts; EW, Emotional work; JI, Job insecurity; PP, Psychosomatic Problems; JS, Job satisfaction; WE, Work engagement; R, resources; M, measures; INF, Information; IMP, impact;

* p < 0.05;

** *p < 0.01*.

## Discussion and Conclusions

This article deals with an issue that is rarely addressed in the scientific literature: the interaction between the psychosocial risks faced by health professionals, specifically nurses, during a health crisis such as the pandemic generated by COVID-19. The impact that the virus has had at all levels around the world is enormous (50). It has posed and still poses a challenge in terms of health, economics, politics, and society, as well as an enormous individual and collective effort, where the emotional toll on the general population is significant and prolonged (62). It is a challenge that we are facing as humanity, as a society, and as individuals. Many professionals are working with substantial hourly loads and extreme conditions in this context of incredible demands and many uncertainties, and social and physical overload (63). Among them, the nurses' work is invaluable (47). Any information that we can provide to alleviate as much as possible the heavy physical and psychological burden to which they are being subjected, both at present and on future occasions that we hope will not be repeated for many years, will be an effort well-invested. An effort to take care of caregivers, especially in the extreme crisis of a pandemic.

This study focuses on nurses in Spain, at the peak of the pandemic in this country. The main results of the study show, on the one hand, that nurses in general feel that they have to do a lot of emotional work and that they have a heavy workload, highlighting these two psychosocial risks above all others. This result can be explained by the remarkable effort not to show their emotions. Despite the situation of being exposed to the suffering of patients together with the scarcity of resources and the large amount of worked hours represents a strong emotional strain, nurses feel that they cannot show their emotional state, and they try to offer their best face (11, 64). This situation represents a significant added effort for them and, at the same time, shows their ethical practice, respect for human dignity, human rights, and cultural diversity (65). Also, nurses are expected to provide holistic care from a cultural, environmental, social, psychological, economic, and spiritual perspective (66). On the other hand, the psychosocial risk that has received the lowest scores is psychosomatic problems. Perhaps this could be due to the pandemic's peak situation; nurses have not yet developed physical symptoms that are the product of the psychological wear to which they are subjected, and that later is when psychosomatic symptoms are likely to emerge (23).

Regarding job satisfaction and work engagement, they tend to be high, which speaks to a certain resilience in the participants, perhaps due to the awareness of the enormous importance of the work to be done, especially and more than ever in these extreme circumstances. Studies have identified that nurses were able to manage their vulnerability using their strengths (personal, professional, contextual, and spiritual) by increasing their resilience. These strengths reflected a balance of personal attributes such as personal values (caring), attitudes (being optimistic), beliefs (religion) along with their professional skills (communication) in the contexts in which they worked (work environment, available support) (67). Resilient nurses are more likely to remain in the workforce (68), which is of vital concern due to the international COVID- 19 crisis. In this context, nurses consider the impact of the COVID-19 on their work to be high, although it does not obtain the maximum score. This outcome could be because the questions refer to the work in particular, and yet the COVID-19 has strongly impacted all spheres of society worldwide, affecting personal, family, and social relationships in general. This fact could lead nurses to consider the impact of COVID-19 not as a particular impact on their workplace, but as a general impact that goes far beyond (50, 62).

In relation to the perception of the measures taken by the responsible entities, as well as the resources and information available, it is considered by nurses to be of a medium level, being neither especially good nor bad. A possible explanation to this could be that they value both the positive and negative aspects, and make an average between what they perceive to be good, and what they perceive to be not so good. Nurses who received frequent and evidence-based information from hospital management expressed less anxiety about the pandemic. Concern for one's own health and the health of the family requires accurate, timely, and frequent communication from healthcare managers and experts (69). As for the relationships observed between the variables, most are those expected based on the scientific literature, such as the case of role conflict, workload, and interpersonal conflicts being positively related. The most frequently identified sources of conflict include lack of emotional intelligence, personality traits, various aspects of the job and work environment, role ambiguity, lack of support from manager and colleagues, and poor communication (70). The data suggest that job satisfaction is inversely related to these psychosocial risks (role conflict and interpersonal conflict) and to psychosomatic problems (71).

Also, in line with expectations based on previous research, the measures, resources, and information related to COVID-19 are related to each other, while the impact of COVID-19 seems to be independent of them. Interestingly, the COVID-19 measures, resources, and information relate to increased job satisfaction, which supports the theory that the more resources available to address job challenges, the greater the satisfaction and less the discomfort associated with the job (22). On the other hand, also in line with what is expected based on the scientific literature, more resources, measures, and information appear to be related to less conflict of roles, which could indicate that these measures, resources, and information facilitate the fact that nurses perceive fewer discrepancies in terms of what is expected of them, having in turn less interpersonal conflicts, greater job satisfaction, and less psychosomatic problems.

Contrary to what might be expected, the psychosocial risks and their associated consequences during the pandemic do not seem so severe, despite the pandemic's difficulties, the overload of work, and the increase in demands of all kinds, including emotional ones. These findings may reflect the nursing staff's character, who, in crises, focus on the care of patients, ignoring the problems, or difficulties of their working conditions. Probably once the crisis is over, nurses will assess somewhat more objectively and also more negatively the conditions in which they had to perform their work.

One possible limitation of the present study refers to the small number of participants, and the short period in which the data was collected. Despite these limitations, we consider the information collected to be extremely valuable, as it collects data on nurses' perceptions of demands, and resources right during the peak of the pandemic, which gives much validity to their responses. The data has not been collected *a posteriori*, where other variables can contaminate the data at that time, such as memory, change of situation, among others. We are also aware of the limitations that this study poses in terms of its results, since it is cross-sectional and no causal relationships can be established between the variables. However, we believe that the data provided are valid and relevant. We hope that they will contribute to better help nurses and health professionals in general in future health crisis situations, especially taking into account such essential elements in the prevention of future pathologies psychosocial risks.

Nurses have played a key role as part of teams managing epidemics threat to health worldwide, (SARS) in 2003 (72), the Middle East Respiratory Coronavirus (MERS-CoV) in 2015 (73), Zika viral disease in 2016 (74, 75), Ebola viral disease in 2014 (76, 77) and the COVID-19 outbreak that began in 2019. Nurses and other health professionals are trained to support their countries' responsiveness to future disasters and emergencies (78). This fact may be particularly important for increasing the resistance of health systems made most vulnerable through disasters and conflict (79).

Finally, it is essential to highlight the significant implications that the data from this study may have for those responsible for taking measures to deal with a pandemic, and for providing the necessary resources and information to health professionals and society in general, in order to prevent the development of multiple pathologies. Our data reflect the importance of the perception of these resources and the information available to face the challenges and demands of a health crisis. These elements can be crucial in ensuring that, despite the heavy workload and the demands that it entails, nurses and health professionals, in general, can perceive satisfaction in what they do, which is a protective factor in the face of physical and psychological pathologies. We believe that studying these relationships is relevant and fundamental to protecting and caring for nurses, health professionals, and society in general.

## Data Availability Statement

The raw data supporting the conclusions of this article will be made available by the authors, without undue reservation.

## Ethics Statement

The studies involving human participants were reviewed and approved by Ethical Committee of Research with Medicines CEIM Code 128/19 Dr. Peset University Hospital. The patients/participants provided their written informed consent to participate in this study.

## Author Contributions

MCG-E, VP-G, and AS-R made a substantial contribution to the concept and design of the work, acquisition, analysis and interpretation of data, drafted the article and revised it critically for important intellectual content, approved the version to be published, and have participated sufficiently in the work to take public responsibility for appropriate portions of the content. All authors contributed to the article and approved the submitted version.

## Conflict of Interest

The authors declare that the research was conducted in the absence of any commercial or financial relationships that could be construed as a potential conflict of interest.
